# Human Conventional and Plasmacytoid Dendritic Cells Differ in Their Ability to Respond to *Saccharomyces cerevisiae*

**DOI:** 10.3389/fimmu.2022.850404

**Published:** 2022-05-11

**Authors:** Andrea Sabatini, Gisella Guerrera, Marta Corsetti, Gabriella Ruocco, Marco De Bardi, Sonia Renzi, Duccio Cavalieri, Luca Battistini, Daniela Francesca Angelini, Elisabetta Volpe

**Affiliations:** ^1^ Molecular Neuroimmunology Unit, Istituto di Ricovero e Cura a Carattere Scientifico (IRCCS) Fondazione Santa Lucia, Rome, Italy; ^2^ Department of Biology and Biotechnology Charles Darwin, Sapienza University, Rome, Italy; ^3^ Neuroimmunology Unit, Istituto di Ricovero e Cura a Carattere Scientifico (IRCCS) Santa Lucia Foundation, Rome, Italy; ^4^ Department of Biology, University of Florence, Florence, Italy

**Keywords:** microbiota, dendritic cells, *Saccharomyces cerevisiae*, T helper polarization, fungi

## Abstract

*Saccharomyces cerevisiae* is a commensal yeast colonizer of mucosal surfaces and an emerging opportunistic pathogen in the mucosa and bloodstream. The role of *S. cerevisiae* has been largely characterized in peripheral blood mononuclear cells and monocyte-derived dendritic cells, where yeast cells induce the production of inflammatory cytokines through the interaction with mannose receptors, chitin receptors, DC SIGN, and dectin1. However, the response of blood-circulating dendritic cells (DCs) to *S. cerevisiae* has never been investigated. Among blood DCs, conventional DCs (cDCs) are producers of inflammatory cytokines, while plasmacytoid DCs (pDCs) are a specialized population producing a large amount of interferon (IFN)-α, which is involved in the antiviral immune response. Here we report that both human DC subsets are able to sense *S. cerevisiae*. In particular, cDCs produce interleukin (IL)-6, express activation markers, and promotes T helper 17 cell polarization in response to yeasts, behaving similarly to monocyte-derived DCs as previously described. Interestingly, pDCs, not cDCs, sense fungal nucleic acids, leading to the generation of P1-pDCs (PD-L1^+^CD80^–^), a pDC subset characterized by the production of IFN-α and the induction of a Th profile producing IL-10. These results highlight a novel role of pDCs in response to *S. cerevisiae* that could be important for the regulation of the host microbiota–immune system balance and of anti-fungal immune response.

## Introduction

In the last years, a crucial role of microbiota is emerging in the development of pathologies involving the immune system, such as allergies, inflammatory disorders, and autoimmune diseases.

Microbiota is the set of microbes, including viruses, bacteria, and fungi, living in the host organism. Microbiota is essential for the protection against pathogens, the synthesis of molecules, and the metabolism of substances derived from diet (1). In a healthy condition, all of these microbes are balanced with each other and are able to induce immune tolerance. However, an alteration in the number and/or localization of different microbes (dysbiosis) may promote diseases associated to immune dysregulation. The microbiota influences the immune system through the interaction with innate immune cells (2). In particular, dendritic cells (DCs) interact with microorganisms through a set of pattern recognition receptors, such as Toll-like receptor (TLR), and initiate the adaptive immune response by activating naïve T lymphocytes through the release of cytokines and the expression of co-stimulatory molecules (3). Blood DCs can be divided into conventional (cDCs) and plasmacytoid DCs (pDCs) (4, 5). pDCs predominantly recognize viruses and are IFN-α producers (6–9), while cDCs are involved in the recognition of several microorganisms, including bacteria and viruses, and produce inflammatory cytokines, such as interleukin (IL)-6 (10).

However, bacteria and viruses are not the unique microbes composing the microbiota. The fungi kingdom is an important component of the microbiota, called mycobiota. Interestingly, the composition of the mycobiota is particularly unstable compared with the rest of the microbiota (11). Thus, fungi derived from dietary or environmental sources may contribute to mycobiota diversity and may strongly influence innate immunity. The most common genus of fungi originating from food and environment and harboring the gastrointestinal tract is *Saccharomyces.* Many members of this genus are considered very important in food production, such as *Saccharomyces cerevisiae*, the bakers’ and brewers’ yeast (11).

However, *S. cerevisiae* is also an etiologic agent of opportunistic fungal infection in immunocompromised patients (12), and the ability to colonize and give rise to disease depends on the host immune response. Although immune response to *Aspergillus fumigatus*, *Candida albicans*, *Cryptococcus neoformans*, and *Malassezia* have been largely characterized (13), little is known about the interactions between *S. cerevisiae* and host defense cells. Previous studies reported that the wall components of *S. cerevisiae* activate human monocyte-derived DCs through mannose receptor, DC SIGN, dectin-1, and chitin receptor, thus leading to the production of IL-6 and other inflammatory cytokines (14–16). However, the ability of blood-circulating human DCs to respond to *S. cerevisiae* has never been investigated. Here we challenged human blood DC subtypes with *S. cerevisiae*, and we found a differential response of cDCs and pDCs, characterized by IL-6 and IFN-α production, with a consequent IL-17 and IL-10 induction in *in vitro*-differentiated Th cells, respectively. Thus, the exposition of *S. cerevisiae* as well as the prevalence of specific DC targets *in situ* may influence the development and the progression of diseases associated to microbiota-dependent immune dysregulation or fungal opportunistic infection.

## Results

### The Laboratory Strain of *Saccharomyces cerevisiae* SK-1 Promotes Activation of Human Blood DCs

**Figure 1:**
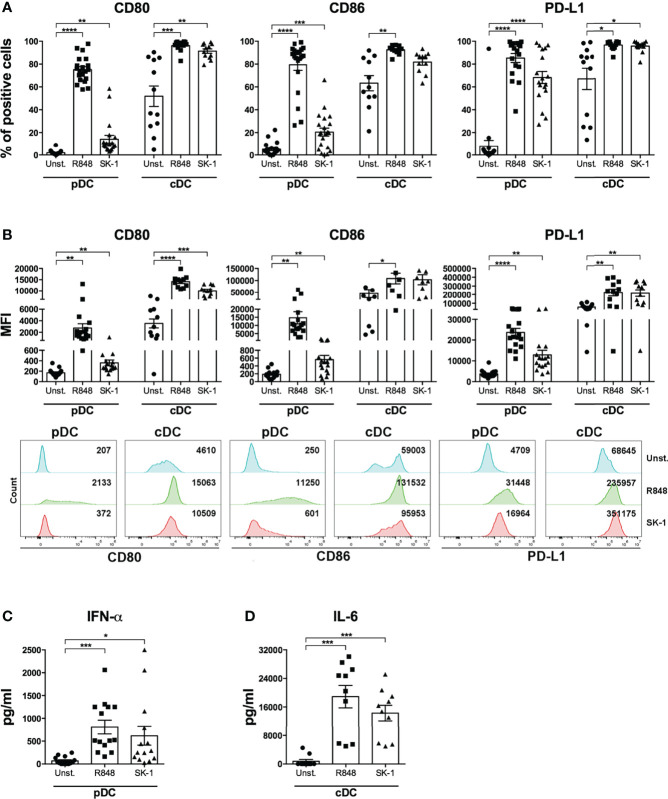
The laboratory strain of *Saccharomyces cerevisiae* SK-1 promotes the activation of human blood dendritic cells. Human pDCs and cDCs purified from the peripheral blood of healthy donors were cultured for 48 h without stimulation (Unst.), with R848 (1 μg/ml) as positive control, or with the laboratory strain of *Saccharomyces cerevisiae* SK-1 at multiplicity of infection = 5 (colony-forming unit SK-1/DC). The expression of molecules CD80, CD86, and PD-L1 was analyzed by flow cytometry, and the percentage of positive cells **(A)** and median fluorescence intensity **(B)** were reported. The levels of IFN-α **(C)** and IL-6 **(D)** were measured in the culture supernatants by ELISA. Data are the mean of 11 independent experiments, each from different donors. Error bars represent SEM. One-way ANOVA was used to compare the different experimental conditions (*=*p*-value ≤ 0.05; **=*p*-value ≤ 0.01; ***=*p*-value ≤ 0.001; ****=*p*-value ≤ 0.0001).

In order to test blood DCs’ response to *S. cerevisiae*, human pDCs and cDCs were purified from the peripheral blood of healthy donors (**Supplementary Figures S1**, **S2**) and stimulated with a laboratory strain of *S. cerevisiae*, called SK-1. Specifically, we cultured DCs in the presence of SK-1 at multiplicity of infection (MOI) of 5 (colony-forming unit SK-1/DC). We used as positive control Resiquimod (R848), which is an imidazoquinoline compound known to be a potent immune activator of both pDCs and cDCs because it is an agonist of TLR7 and TLR8, expressed by pDCs and cDCs, respectively. Unstimulated DCs were used as negative control. We evaluated the levels of CD80 and CD86, two molecules binding CD28 on T cell surface and inducing T cell activation and proliferation, and the expression of programmed death-ligand 1 (PD-L1), which is a co-inhibitory molecule known to reduce T cell proliferation through the binding with PD-1 on T cell surface. Our results showed that SK-1 induces a significant increase of CD80 and CD86 expression by pDCs and cDCs (**Figure 1A**). Surprisingly, we also found a significant increase of PD-L1 expression induced by SK-1 in both DC subsets, especially in pDCs (**Figure 1A**). Consistently, the increase of CD80^+^, CD86^+^, and PD-L1^+^ cells in DCs is associated with an overall high median fluorescence intensity (MFI) (**Figure 1B**). In addition, we measured IFN-α and IL-6 production by SK-1-stimulated pDCs and cDCs, respectively. We found that SK-1 induces IFN-α production by pDCs (**Figure 1C**) and IL-6 production by cDCs (**Figure 1D**) compared with unstimulated DCs.

### SK-1 Induces the Differentiation of P1 Subpopulation of Human pDCs

**Figure 2:**
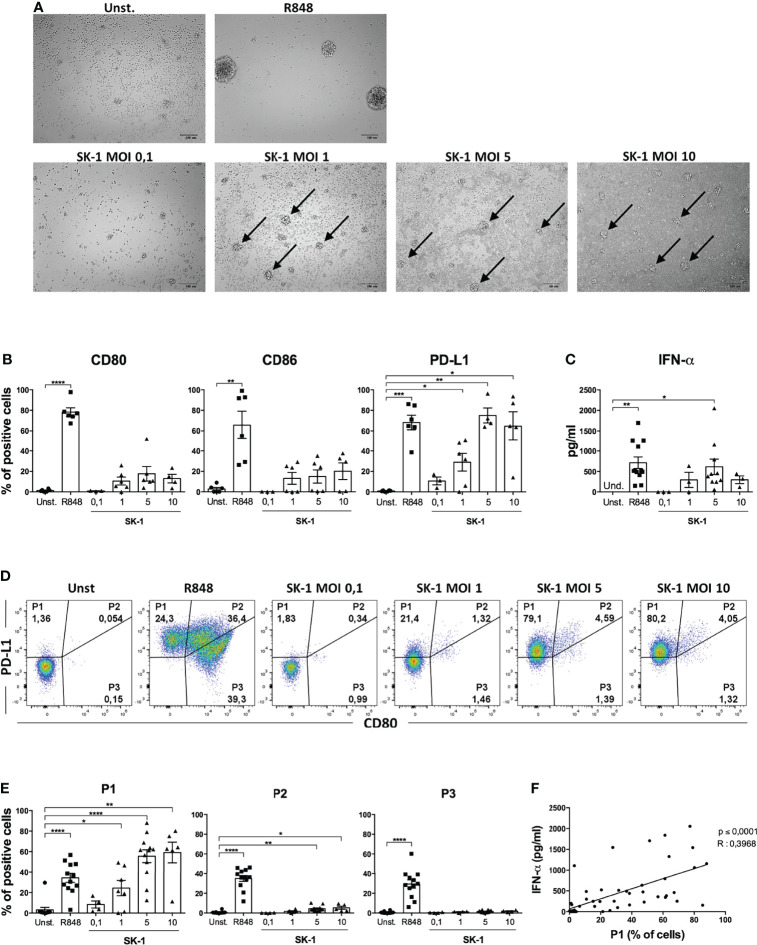
SK-1 induces the differentiation of the P1 subpopulation of human plasmacytoid dendritic cells (pDCs). Human pDCs purified from the peripheral blood of healthy donors were cultured for 48 h without stimulation (Unst.), with R848 (1 μg/ml) as positive control, or with the laboratory strain of *Saccharomyces cerevisiae* SK-1 at different multiplicity of infection values (0.1–1–5–10; colony-forming unit SK-1/pDC). Photos by optical microscopy show cell clusters, indicated by arrows, formed by pDCs upon activation. The pictures are representative of three independent experiments, each from a different donor **(A)**. The expression of molecules CD80, CD86, and PD-L1 was analyzed by flow cytometry and reported as the percentage of positive cells **(B)**. Levels of IFN-α were measured in the culture supernatants by ELISA assay **(C)**. The expression of PD-L1 and CD80 was analyzed by flow cytometry. Representative plots show the percentage of three pDC subpopulations **(D)**, and the graphs show the corresponding quantification of more experiments **(E)**. Data in **(B**, **C**, **E)** are the mean of six or more independent experiments, each from different donors. Error bars represent SEM. One-way ANOVA was used to compare different experimental conditions (*=*p*-value ≤ 0.05; **=*p*-value ≤ 0.01; ***=*p*-value ≤ 0.001; ****=*p*-value ≤ 0.0001). The percentages of P1 subpopulation, obtained from all experimental conditions of 4 independent experiments, were correlated to their IFN-α levels using Pearson correlation **(F)**. The *R* value indicates the correlation coefficient.

To further gain insight into pDC activation by SK-1, we cultured pDCs in the presence of SK-1 at different MOI values. After 48 h of culture, we firstly evaluated the formation of cell clusters, which reflects pDC activation and pDC viability by optical microscopy. Our results showed that stimulation with SK-1 leads to cell cluster formation (**Figure 2A**). As expected, pDCs stimulated with the positive control (R848) form cell clusters, whereas unstimulated pDCs do not form any cell cluster (**Figure 2A**). Moreover, we found that different doses of SK-1 lead to a progressive increase of CD80, CD86, and PD-L1 within viable pDCs in a dose-dependent manner (**Figure 2B**). We found that SK-1 at MOI 5, not at MOI 10, induces the highest levels of IFN-α (**Figure 2C**), which is likely due to the lethal effect of a high dose of SK-1 (data not shown). These results confirm that SK-1 (MOI 5) promotes pDC activation and viability with the concomitant IFN-α production.

Recently, it has been observed that the differential expression of the surface molecules PD-L1 and CD80 defines three specific pDC subpopulations with distinct functions: P1 (PD-L1^+^ CD80^-^), P2 (PD-L1^+^ CD80^+^), and P3 (PD-L1^-^ CD80^+^) (17). Given the high expression of PD-L1 induced by SK-1 in pDCs, we hypothesized that the P1-pDC subpopulation is preferentially induced upon stimulation with the laboratory strain of *S. cerevisiae*. Thus, we analyzed pDC subpopulations in our experimental conditions by flow cytometry. Our results showed that SK-1 is able to induce P1-pDC subpopulation (**Figures 2D, E**). In particular, we observed a dose-dependent induction of the percentage of P1-pDCs at increasing doses of SK-1. The positive control R848 induces all three subpopulations (**Figures 2D, E**), as previously demonstrated (17). The fluorescence-minus-one experiment demonstrates the specificity of PD-L1 staining (**Supplementary Figure S3**).

Moreover, it has been reported that IFN-α-producing pDCs are mostly P1-pDCs (9). Therefore, we investigated whether the induction of P1-pDCs obtained upon stimulation with SK-1 was associated to IFN-α production in the same experimental conditions. To address this relationship, we correlated the levels of IFN-α and the percentage of P1-pDCs in several pDC-SK-1 cultures. Interestingly, we found a positive correlation between P1-pDCs and IFN-α (**Figure 2F**), suggesting that SK-1 induces PD-L1^+^ CD80^-^ pDCs, which, in turn, produce IFN-α.

### Fungal Nucleic Acids Activate Human pDCs

**Figure 3:**
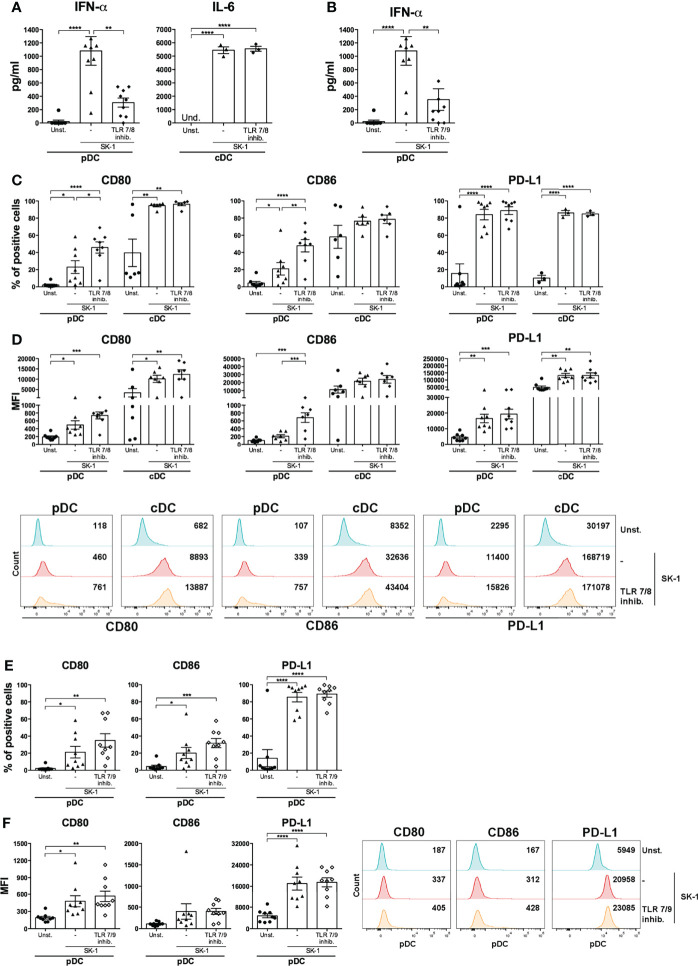
IFN-α production by SK-1-stimulated plasmacytoid dendritic cells (pDCs) is mediated by sensors of nucleic acids. Human pDCs and conventional dendritic cells (cDCs) purified from the peripheral blood of healthy donors were pre-treated for 30 min with TLR 7/8 (1 μM) or with TLR 7/9 (1 μM) and cultured for 48 h without stimulation (Unst.) or with the laboratory strain of *Saccharomyces cerevisiae* SK-1 at multiplicity of infection = 5 (SK-1/DC). The levels of IFN-α or IL-6 were measured in the culture supernatants by ELISA **(A, B)**. The expression of molecules CD80, CD86, and PD-L1 was analyzed by flow cytometry and reported as the percentage of positive cells and median fluorescence intensity **(C–F)**. The graphs show the mean ± SEM of three or more independent experiments, each from different donors. Two-way ANOVA was used to compare different experimental conditions (*=*p*-value ≤ 0.05; **=*p*-value ≤ 0.01; ***=*p*-value ≤ 0.001; ****=*p*-value ≤ 0.0001).

In order to investigate the mechanism inducing blood DC activation by *S. cerevisiae*, we firstly analyzed the involvement of TLR7 and TLR8, able to recognize microbial RNA, in SK-1-mediated DC activation. We performed a set of experiments in the presence of a synthetic antagonist inhibitor of TLR7 in pDCs and TLR8 in cDCs. First, human-purified DCs were pre-treated for 30 min with the inhibitor and then stimulated for 48 h with SK-1 at MOI 5. We analyzed the IFN-α production in pDCs and IL-6 production in cDCs, and we compared the results with those obtained in SK-1-stimulated DCs without inhibitor. Interestingly, IFN-α production in pDCs, not IL-6 in cDCs, is significantly reduced in the presence of TLR7-8 inhibitor (**Figure 3A**). Importantly, we tested different doses of TLR7-8 inhibitor, and the results confirmed that TLR8 is not involved in IL-6 production by cDCs stimulated with SK-1 (**Supplementary Figure S4A**), while it is involved in those stimulated with R848, which is the ligand of TLR7-8 (**Supplementary Figure S4B**). Since pDCs also express TLR9, which recognizes microbial DNA, we cultured SK-1-stimulated pDCs in the presence of TLR7-9 inhibitor. We observed a reduction of IFN-α levels in pDCs stimulated with SK-1 and pre-treated with TLR7-9 inhibitor (**Figure 3B**). However, we could not appreciate an additive effect due to the inhibition of both TLR7 and TLR9 signaling compared with the inhibition of TLR7 alone (**Figure 3B**). In contrast, the expression of CD80, CD86, and PD-L1 seems to be not mediated by fungal nucleic acids. In fact, PD-L1 expression in SK-1-stimulated pDCs and cDCs was not affected by the presence of TLR7-8-9 inhibitors (**Figures 3C–F**), and the CD80 and CD86 expression, respectively, were weakly increased in pDCs in the presence of TLR7-8 inhibitor (**Figures 3C, D**). These results suggest that IFN-α production in pDCs could be mediated by the interaction between yeast nucleic acids and TLR, while the upregulation of surface markers on the pDC surface depends on other pathways, which are further activated in response to TLR7 inhibition. On the other hand, recognition of whole SK-1 by cDCs occurs through other pathways independent of TLR8.

**Figure 4:**
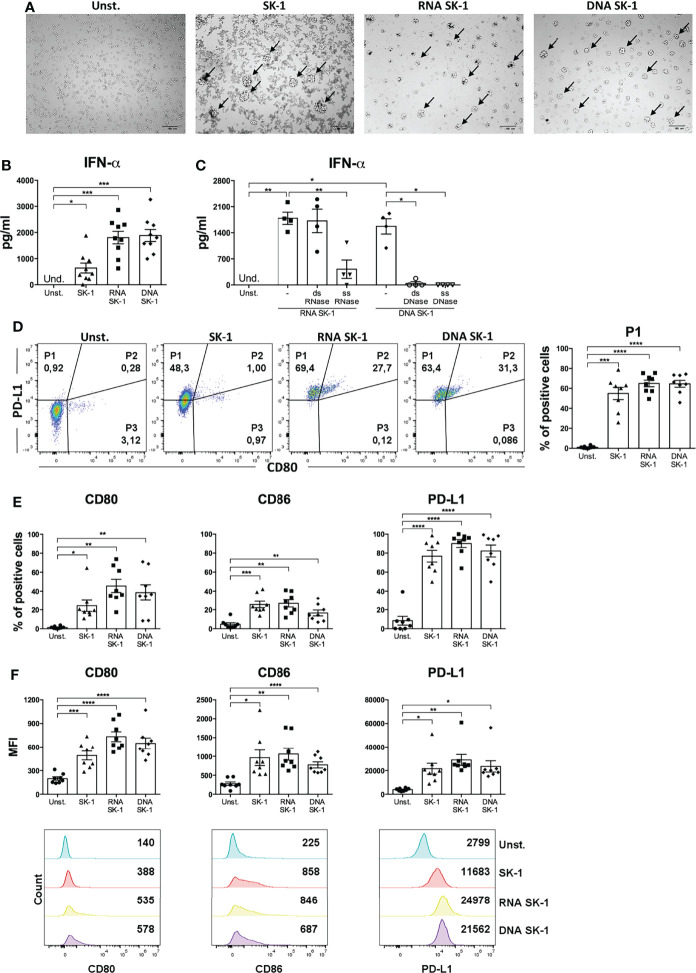
RNA and DNA of SK-1 induce activation of human plasmacytoid dendritic cells (pDCs). Human pDCs purified from the peripheral blood of healthy donors were cultured for 48 h with RNA or DNA (0.2 μg) extracted from *Saccharomyces cerevisiae* SK-1 and pre-treated with Dotap (10 μl/μg of nucleic acids) for 30 min at 37°C without stimulation (Unst.) and with the laboratory strain of *Saccharomyces cerevisiae* SK-1 at multiplicity of infection = 5 (SK-1/pDC). Photos taken with an optical microscope show the cell clusters formed by pDCs upon activation highlighted by arrows **(A)**. The pictures are representative of four experiments, each from a different donor. DNA and RNA were pre-treated with nucleases where indicated. The levels of IFN-α were measured in the culture supernatants by ELISA **(B, C)**. The percentage of the P1-pDC subpopulation was evaluated by flow cytometry **(D)**. The percentages and median fluorescence intensity of costimulatory molecules CD80, CD86, and PD-L1 were analyzed by flow cytometry **(E, F)**. The graphs show the mean ± SEM of eight independent experiments, each from different donors. One-way ANOVA was used to compare different experimental conditions (*=*p*-value ≤ 0.05; **=*p*-value ≤ 0.01; ***=*p*-value ≤ 0.001; ****=*p*-value ≤ 0.0001).

In order to directly assess whether *S. cerevisiae* nuclei acids induce IFN-α production by pDCs, we stimulated blood pDCs with RNA and DNA extracted from SK-1. We found that both nucleic acids induce cell cluster formation typical of pDC activation (**Figure 4A**) and IFN-α production (**Figure 4B**). To further characterize this response, we performed experiments in the presence of nucleases specifically degrading single-strand (ss) or double-strand (ds) yeast nucleic acids. We found that degradation of ssRNA, ssDNA, and dsDNA of SK-1 nucleic acids inhibits IFN-α production by human pDCs (**Figure 4C**).

**Figure 5:**
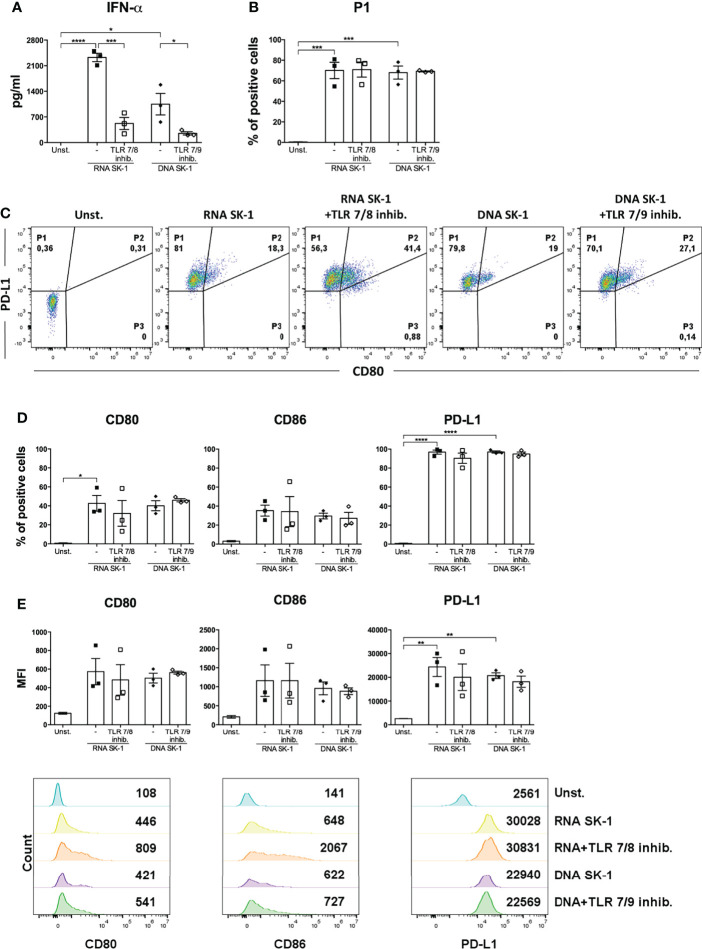
IFN-α production by human plasmacytoid dendritic cells (pDCs) stimulated with SK-1 nucleic acids is partially mediated by TLR7 and TLR9. Human pDCs purified from the peripheral blood of healthy donors were pre-treated for 30 min with TLR7/8 (1 μM) or with TLR7/9 (1 μM) and cultured for 48 h with RNA or DNA (0.2 μg) after incubation with Dotap (10 μl/μg of nucleic acids) for 30 min at 37°C without stimulation (Unst.). The levels of IFN-α were measured in the culture supernatants by ELISA **(A)**. The percentage of the P1-pDC subpopulation was evaluated by flow cytometry **(B, C)**. The percentages and median fluorescence intensity of costimulatory molecules CD80, CD86, and PD-L1 were analyzed by flow cytometry **(D, E)**. The graphs show the mean ± SEM of three independent experiments, each from different donors. Two-way ANOVA was used to compare different experimental conditions (*=*p*-value ≤ 0.05; **=*p*-value ≤ 0.01; ***=*p*-value ≤ 0.001; ****=*p*-value ≤ 0.0001).

Interestingly, nucleic acids from SK-1 do not induce the production of pro-inflammatory cytokine IL-6 by cDCs (**Supplementary Figure S5**). Moreover, RNA and DNA from SK-1 induce P1-pDC differentiation (**Figure 4D**), upregulation of the activation markers (CD80 and CD86), and the inhibitory marker PD-L1 at a similar extent to whole SK-1 (**Figures 4E, F**). These data collectively indicate that yeast nucleic acids specifically activate human pDCs, leading to IFN-α, CD80, CD86, and PD-L1 overexpression. In order to investigate the role of TLRs in recognizing SK-1 nucleic acid in pDCs, we used TLR inhibitors in yeast DNA- and yeast RNA-treated cells, and we found that TLR7 and TLR7/9 inhibitors partially reduce IFN-α production by SK-1 RNA and DNA, respectively (**Figure 5A**). The percentage of P1-pDC subpopulation (**Figures 5B, C**) and the expression of CD80, CD86, and PD-L1 are not affected by the presence of TLR7 and TLR9 inhibitors (**Figures 5D, E**).

### SK-1-Primed pDCs and cDCs Induce IL-10- and IL-17-Producing CD4 T Cells

Activated DCs perform important adaptive functions in naïve CD4 T cell priming and polarization. Given the differential response of pDCs and cDCs to *S. cerevisiae* in terms of cytokine production and costimulatory molecule expression, we hypothesized a functional specialization towards distinct T helper (Th) profiles. We stimulated naïve CD4 T cells with anti-CD3 to simulate antigen recognition and with SK-1-primed DCs to study the impact of the upregulation of their cytokines and co-stimulatory receptors on Th polarization. We analyzed the production of typical Th cytokines (IFN-γ for Th1, IL-4 for Th2, IL-17 for Th17, and IL-10 for T regulatory cells) in T cells co-cultured with SK-1-primed DCs compared with T cells co-cultured with unstimulated DCs and T cells stimulated with anti-CD3 and anti-CD28.

**Figure 6:**
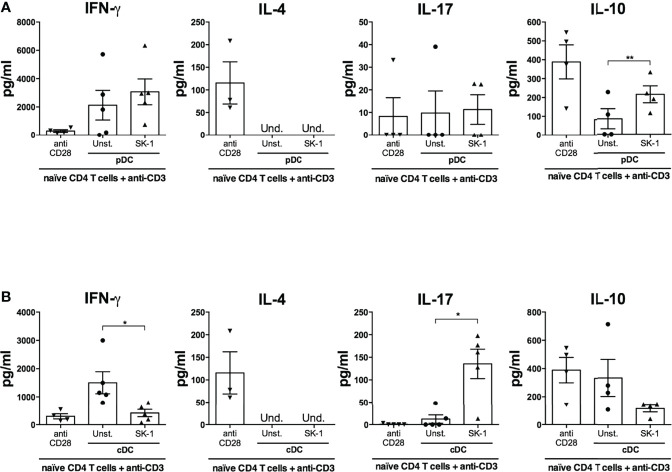
Plasmacytoid dendritic cells (pDCs) and conventional dendritic cells (cDCs) primed with SK-1 induce IL-10- and IL-17-producing CD4 T cells, respectively. Human naïve CD4 T cells were cultured in the presence of anti-CD3/CD28 beads or placed in a co-culture for 5 days with unstimulated or SK-1-primed pDCs or cDCs in the presence of anti-CD3. After 5 days, the cells were re-stimulated with anti-CD3 (co-culture) or anti-CD3/CD28 beads (CD4 T cells alone). Supernatants were collected after 24 h of re-stimulation, and the levels of IFN-γ, IL-4, IL-17, and IL-10 in co-cultures with pDCs **(A)** and cDCs **(B)** were measured by ELISA. The graphs show the mean ± SEM of four or five independent experiments, each from different donors. Paired Student’s *t*-test was used to compare unstimulated and stimulated DCs (*=*p*-value ≤ 0.05; **=*p*-value ≤ 0.01).

We found that IL-4 is not significantly modulated in the different experimental conditions (**Figures 6A, B**), while SK-1-primed cDCs induce significant IL-17 production compared with unstimulated cDCs (**Figure 6B**) and SK-1-primed pDCs induce significant IL-10 production compared with unstimulated pDCs (**Figure 6A**). Concomitantly to IL-17 production, we observed a significant reduction of IFN-γ by naïve CD4 T cells co-cultured with SK-1-primed cDCs (**Figure 6B**).

## Materials and Methods

### Purification of DC Subpopulations (cDCs and pDCs) From Adult Blood

Peripheral blood mononuclear cells (PBMCs) were isolated by Ficoll gradient centrifugation (GE Healthcare) from the whole blood of healthy donors (Santa Lucia Foundation, Rome, Italy).

Approval by the ethics committee of the Santa Lucia Foundation, Rome (Italy), and written informed consent in accordance with the Declaration of Helsinki from all participants were obtained before the study was initiated. PBMCs (400 × 10^6^) were enriched using the Human Pan-DC Pre-Enrichment Kit (Stemcell Technologies), specific for the purification of all DC type, by negative selection. After isolation, the cells were stained with the following antibodies: anti-human CD11c PE-Dazzle594 (BD Biosciences), anti-human CD4 PC7 (Beckman Coulter), anti-human CD3 PE (Beckman Coulter), anti-human CD14 PE (Immunological Science), anti-human CD16 PE (Miltenyi Biotec), anti-human CD56 PE (Beckman Coulter), anti-human CD19 PE (Miltenyi Biotec), and anti-human CD235α (eBioscience).

Highly purified pDC and cDC cells were selected and sorted by 6-way sorter MoFlo Astrios (Beckman Coulter) by using the following analysis as previously described (18): lineage (CD3, CD14, CD16, CD56, CD19, CD235α)^-^, CD4^+^, CD11c^-^ for pDCs and lineage (CD3, CD14, CD16, CD56, CD19, CD235α)^-^, CD4^+^, CD11c^+^ for cDCs. The sorted cells had a purity of over 95 and 94%, respectively, for pDCs and cDCs, as confirmed by flow cytometry analysis (**Supplementary Figures S1A, B**). This gating strategy includes only pDCs CD123^+^ and cDCs HLA-DR^+^ cells (**Supplementary Figure S2**).

### *In Vitro* Stimulation of pDCs and cDCs

pDCs and cDCs were cultured separately in 96-well flat-bottom half-area plates (Corning) at a density of 5 × 10^4^ per well in RPMI 1640 with 5% of human serum for 48 h at 37°C with 5% of CO_2_, in the absence of stimuli or in the presence of R848 (InvivoGen; 100 ng/ml), SK-1 (*Saccharomyces cerevisiae*; MOI: 0.1–1–5–10 SK-1 cells/cDC or pDC). For the SK-1 nucleic acid stimulation experiments, the RNA and DNA (0.2 μg) were extracted from SK-1 as previously described (16), and their quality and purity were verified by agarose gel electrophoresis and using a Nanodrop 2000 spectrophotometer (Thermofisher; **Supplementary Figure S6**). SK-1 RNA and SK-1 DNA samples were used for pDC stimulation pre-treated with Dotap (Merck; 10 μl/μg of nucleic acids). Pre-treatment of nucleic acids with ezDNase or RNase III (Thermofisher) was performed to degrade dsDNA and dsRNA, respectively, while S1 nuclease (Thermofisher) was used to degrade ssDNA and ssRNA. For the TLR inhibition experiments, cDCs and pDCs were pre-treated for 30 min with TLR7/8 (Miltenyi Biotec ODN 2087) or TLR7/9 (Miltenyi Biotec ODN 2088) inhibitors (1 μM) and then stimulated with SK-1 (MOI 5).

### Naïve CD4 T Cells and DC Subset Coculture

pDC and cDC subsets from healthy donors were cultured separately in 96-well flat-bottom half-area plates (Corning) at a density of 5 × 10^4^ per well in RPMI 1640 with 5% of human serum in the absence of stimuli or in the presence of SK-1 (MOI 5). CD4 T lymphocytes were purified from the PBMCs of the same healthy donors by immunomagnetic selection using anti-Mouse IgG MicroBeads (Miltenyi Biotec). After isolation, the cells were stained with anti-human CD4 PC7 (Beckman Coulter), anti-human CD45RA BV421 (BD Biosciences), anti-human CD45RO PE (BD Biosciences), and anti-human CD27 APC (Beckman Coulter), and CD4 naïve T cells were sorted by Astrios high-speed cell sorter (Beckman Coulter) as CD4^high^, CD45RA^high^, CD45RO^-^, and CD27^+^. The sorted cells had a purity of over 97%, as shown by flow cytometry (**Supplementary Figure S7**).

After 24 h, the stimulated DCs were collected and cocultured with naive CD4 T cells at a density of 5 × 10^4^ DCs and 5 × 10^4^ lymphocytes (ratio 1:1) per well in X-VIVO 15 serum-free medium (Lonza) in 96-well round-bottom plates (Falcon) coated with anti-CD3 (BD Biosciences; 10 μg/ml). Naïve CD4 T cells stimulated with Dynabeads CD3/CD28 T cell expander (1 bead per cell; Life Technologies) were used as control. After 5 days, the cells were harvested and washed, and viability was determined by Trypan Blue exclusion. Then, the cells were resuspended in X-VIVO 15 serum-free medium (Lonza) at a concentration of 1 × 10^6^ cells/ml and restimulated with anti-CD3 for 24 h in 96-well flat-bottom plates (Falcon). The conditions of incubation were stable (temperature at 37°C with 5% CO_2_).

### Flow Cytometry Analysis

pDCs and cDCs were harvested after 48 h of culture, resuspended in an EDTA-containing medium, and then stained for 15 min at 4°C with the following antibodies: anti-human CD4 PC7 (Beckman Coulter), anti-human PD-L1 PE (Biolegend), anti-human CD86 APC (Miltenyi Biotec), and anti-human CD80 BV650 (BD Bioscience). The samples were washed in EDTA-containing medium, acquired using CytoFLEX cytometer (Beckman Coulter) and analyzed by FlowJo-10 software (version 10.3.0).

### Analysis of Cytokine Production

Cytokines were measured in supernatants from pDC and cDC cultures, respectively, using IFN-α ELISA (Invitrogen, Human IFN alpha Antibody Pair Kit), IL-6 ELISA (R&D Systems, Human IL-6), IL-17 ELISA (R&D Systems, Human IL-17), IL-4 ELISA (R&D Systems, Human IL-4), IFN-γ ELISA (R&D Systems, Human IFN-γ), and IL-10 ELISA (R&D Systems, Human IL-10) according to the manufacturer’s instructions.

### Statistical Analysis

Statistical analyses were performed using one-way ANOVA, two-way ANOVA, or Student’s *t*-test, depending on the number of experimental conditions and independent variables. We used GraphPad Prism software (version 6.01, GraphPad Software). Data were presented as mean ± standard error (SEM). The *p*-values of 0.05 or less were considered statistically significant.

## Discussion

SK-1 is a fungus with phylogenetic similarity to yeasts isolated from fermentation in the African area (19) and is most likely one of the constituents of the human microbiota derived from the ingestion of fermentation products. However, *S. cerevisiae* has also been found in the bloodstream of immunocompromised patients (20–22), thus considering this fungus an emerging opportunistic pathogen (23). Therefore, our study is useful for understanding the interaction of mucosal DCs with intestinal microbiota and for the analysis of the anti-fungal immunity of blood DCs against pathogenic *S. cerevisiae*. The results from this study revealed that SK-1 is able to activate human blood pDCs and cDCs, indicating that both DC subsets have an important role in modulating the immune response following a fungal stimulus. Importantly, we observed a differential activation of pDCs and cDCs by SK-1, suggesting a distinct role of each DC subset in the anti-fungal immunity and in the interaction with intestinal microbiota.

Specifically, SK-1 induces a high induction of IFN-α and expansion of a subpopulation of pDCs, called P1, characterized by the expression of PD-L1 molecule on the cell surface. However, SK-1 does not stimulate a strong maturation process in these cells; in fact, the co-stimulatory molecules CD80 and CD86 are only weakly induced, unlike what happens as a consequence of their activation mediated by viruses (24), which, in our system, is simulated by R848. A robust IFN-α production associated to a weak induction of costimulatory molecules at the pDC surface is a typical response of CpG-A (25). Thus, similarly to CpG-A, fungal nucleic acids could form a large multimeric complex upon internalization that is retained in early endosomes and signals through MyD88 and IRF-7 (26).

We observed that SK-1 promotes the expansion of P1-pDC subpopulation (PD-L1^+^ CD80^-^), which is specialized in the production of IFN-α and in the induction of the anti-inflammatory cytokine IL-10 by T cell lymphocytes (17). Consistently, our data showed that pDCs stimulated with SK-1 induce IL-10-producing T cells. It is known that IL-10 and IFN-α synergistically promote the differentiation of a particular population of regulatory T lymphocytes known as Tr1 (IL-10^+^, IFN-γ+, IL-2^-^/^low^, and IL4^-^), which is capable of suppressing the proliferation of T lymphocytes (27). These results altogether suggest that SK-1, by inducing pDCs to produce IFN-α and the consequent generation of IL-10-producing T cells, may contribute to the generation of immunoregulatory T cells. The activation of a regulatory response by pDCs has been described in previous papers (7, 28–30), suggesting that this is a typical property of pDCs regardless of the nature of the activating stimulus.

Moreover, the immunosuppressive activity of T regulatory cells has been described in fungal infections (31–34). Specifically, the anti-inflammatory role of DCs in response to fungi is mediated by the enzyme indoleamine 2,3-dioxygenase (IDO) (35), which is associated with the induction of IL-10-producing T regulatory cells (33). Thus, the metabolic pathway involving tryptophan catabolism could be involved in pDC response to SK-1 and local tolerogenic responses. Given the importance of the anti-inflammatory responses in chronic autoimmune diseases, such as multiple sclerosis and Crohn’s disease, pDC response to SK-1 could play a protective role in these diseases.

In contrast to pDCs, cDCs express high levels of CD80 and CD86 in response to SK-1, produce high levels of the inflammatory cytokine IL-6, and promote a Th17 response, suggesting that the interaction between *S. cerevisiae* and cDCs in the gut contributes to mucosal inflammation and to mucosal host defense against fungal infection (36). Importantly, cDCs promote a simultaneous reduction of IFN-γ production by CD4 T cells that suggests a prominent role of *S. cerevisiae* and cDCs in regulating the balance between IL-17-producing (Th17) and IL-17/IFNγ-producing (Th1/17) cells.

It is already known that the yeast cell wall, characterized by galactose and glucosamine (chitin moieties), glucose (beta-glucan), and mannose (mannans), induces the inflammatory response in monocyte-derived DCs (14). Our data suggest that the components of the yeast cell wall are also involved in the activation of blood cDCs, cells which share numerous similarities with monocyte-derived DCs.

However, here, for the first time, we reported that the nucleic acids of *S. cerevisiae* specifically induce cell activation and IFN-α production in pDCs. In particular, ssRNA, ssDNA, and dsDNA derived from SK-1 are involved in pDC activation. Importantly, the induction of IFN-α is partially mediated by TLR7 and TLR9, while the upregulation of co-stimulatory molecules in pDCs is mediated by other pathways. Among the potential receptors of yeast PAMPs, we suppose that galectin-3 and Fc-γ receptor, recognizing yeast mannans, and DNA-PK, cGAS (CGAS), MRE11, DHX36, DHX9, DDX41, and DDX60, sensing yeast nuclei acids, may have a role in SK-1-mediated activation because they are expressed by human pDCs (**Supplementary Figures S8A, B**).

We do not observe an additive effect in IFN-α blocking due to the inhibition of both TLR7 and TLR9 signaling compared with the inhibition of TLR7 alone that could be due to a different effectiveness exerted by TLR7/8 and by TLR7/9 inhibitors on TLR7 or to the involvement of other DNA sensors in pDCs. In this context, it has been reported that pDCs express DHX36, DHX9 (37), and cGAS (38) (**Supplementary Figure S8B**), which are TLR9-independent DNA sensors.

Previous studies reported that nucleic acids induce the maturation of DCs and generate an anti-fungal immune response (39, 40). Specifically, DNA is recognized by TLR9 in *C. albicans* (41, 42), *A. fumigatus* (43), and *C. neoformans* (44) infection, while *S. cerevisiae* and *C. albicans* RNA are recognized by TLR7 (40, 45) and *C. albicans* and *A. fumigatus* RNA by MDA5 (39, 40, 46, 47).

Interestingly, our data indicate that TLR8 inhibitor does not reduce IL-6 production nor the upregulation of co-stimulatory molecules in cDCs, indicating that the SK-1 cell wall components are the main stimuli for cDCs and that the response to SK-1 nucleic acids is a typical feature of pDCs.

These results altogether indicate that the exposure to *S. cerevisiae* triggers pro- or anti-inflammatory responses depending on the interaction with cDCs or pDCs, respectively. Importantly, the nucleic acids of *S. cerevisiae* are a specific anti-inflammatory trigger for pDCs. This information indicates that the opposite role of pDCs and cDCs in response to *S. cerevisiae* could mediate the equilibrium between the pro-inflammatory and the anti-inflammatory immune responses in the gut, which is important either for gut homeostasis or during a *S. cerevisiae* opportunistic infection. The immune dysregulation, characterized by an imbalanced prevalence of cDCs or pDCs, could alter this equilibrium and could lead to the development of autoimmune diseases or exacerbation of fungal infections.

Future studies aimed to investigate the molecular mechanisms leading to IFN-α production by pDCs, such as the identification of the activating RNA or DNA sequence of *S. cerevisiae*, could open new perspectives towards therapeutic approaches for dysbiosis-related diseases, such as probiotic intervention. On the other side, the identification of the molecular mechanisms leading to the pro-inflammatory activity of cDCs by *S. cerevisiae* could be useful for the therapeutic targeting of chronic inflammatory diseases and for a better understanding of the mechanisms underlying the immune response during fungal infections in immunocompromised patients.

## Data Availability Statement

The raw data supporting the conclusions of this article will be made available by the authors without undue reservation.

## Ethics Statement

The studies involving human participants were reviewed and approved by the IRCCS Fondazione Santa Lucia. The patients/participants provided their written informed consent to participate in this study.

## Author Contributions

AS and GG performed the research and analyzed the data. MC and GR performed some experiments. MDB performed sorting experiments. SR cultured and provided *S. cerevisiae* and nucleic acids of SK-1. DC and LB contributed to the research design. DFA designed the research and analyzed the data. EV designed the research, analyzed the data, and wrote the paper. All authors contributed to the article and approved the submitted version.

## Funding

This work was partially supported by the “Progetto Giovani Ricercatori” Italian Ministry of Health, Italy (cod. GR-2016-02361163) and FISM-Fondazione Italiana Sclerosi Multipla (cod. FISM2016/R/31; cod. FISM2020/R-Single/007) to EV, by FISM-Fondazione Italiana Sclerosi Multipla (FISM Progetto Speciale 2018/S/5) and Ricerca Corrente 2022 (linea 2) to LB.

## Conflict of Interest

The authors declare that the research was conducted in the absence of any commercial or financial relationships that could be construed as a potential conflict of interest.

## Publisher’s Note

All claims expressed in this article are solely those of the authors and do not necessarily represent those of their affiliated organizations, or those of the publisher, the editors and the reviewers. Any product that may be evaluated in this article, or claim that may be made by its manufacturer, is not guaranteed or endorsed by the publisher.

## Supplementary Material

Supplementary Figure 1 Gating strategy for the purification of blood dendritic cells (DCs). Human peripheral blood mononuclear cells purified from the peripheral blood of healthy donors were enriched using the Human Pan-DC Pre-Enrichment Kit (EasySep) and then labeled with specific antibodies conjugated with a fluorochrome. Using a cell sorter, the plasmacytoid dendritic cells (pDCs) were isolated as lineage (CD3, CD14, CD16, CD19, CD56, CD235α)^-^ CD4^+^, CD11c^-^, while conventional dendritic cells (cDCs) were isolated as lineage (CD3, CD14, CD16, CD19, CD56, CD235α)^-^ CD4^+^, CD11c^+^ **(A)**. The purity of the isolated pDCs and cDCs, evaluated by flow cytometry after purification, is more than 94% **(B)**.

Supplementary Figure 2 The human blood plasmacytoid dendritic cells (pDCs) and conventional dendritic cells (cDCs) used in this study are CD123^+^ and HLA-DR^+^ cells, respectively. Human peripheral blood mononuclear cells purified from the peripheral blood of healthy donors were labeled with specific antibodies conjugated with a fluorochrome. pDCs were identified as lineage (CD3, CD14, CD16, CD19, CD56, CD235α)^-^ CD4^+^, CD11c^-^, while cDCs were identified as lineage (CD3, CD14, CD16, CD19, CD56, CD235α)^-^ CD4^+^, CD11c^+^. Staining of CD123 and HLA-DR before **(A)** and after sorting **(B)** reveals that pDCs are CD123^+^ cells, while cDCs are HLA-DR^+^ cells. The plots show the data of a representative of more experiments.

Supplementary Figure 3 Fluorescence-minus-one control (FMO) for PD-L1 staining. Human plasmacytoid dendritic cells (pDCs) purified from the peripheral blood of healthy donors were cultured for 48 h without stimulation (Unst.), with R848 (1 μg/ml), or with the laboratory strain of *Saccharomyces cerevisiae* SK-1 at multiplicity of infection = 5 (colony-forming unit SK-1/pDC). Cells were stained with anti-CD80 BV650 (FMO control) or anti-PD-L1 PE and anti-CD80 BV650 (PD-L1 staining). Percentage of PD-L1^+^ cells **(A)**, MFI of PD-L1 **(B)**, and percentage of P1, P2, P3 pDC subpopulations **(C)** were represented. Plots and histograms are from one representative of more experiments.

Supplementary Figure 4 Human conventional dendritic cell (cDC) activation by SK-1 is not affected by TLR8. Human cDCs purified from the peripheral blood of healthy donors were cultured for 48 h without stimulation (Unst.) and pre-treated for 30 min with different doses of TLR inhibitor, as indicated, with the laboratory strain of *Saccharomyces cerevisiae* SK-1 at multiplicity of infection = 5 (SK-1/conventional dendritic cells) **(A)**, or with 5 μg/ml TLR8 inhibitor and R848 (0.1 μg/ml) **(B)**. The levels of IL-6 were measured in the culture supernatants by ELISA. The graphs show the mean ± SEM of 3 independent experiments, each from different donors. Two-way ANOVA was used to compare different experimental conditions (**p*-value ≤ 0.05).

Supplementary Figure 5 Nucleic acids from *Saccharomyces cerevisiae* SK-1 do not induce the production of IL-6 by conventional dendritic cells (cDCs). Human cDCs purified from the peripheral blood of healthy donors were cultured for 48 h with RNA or DNA (0.2 μg) extracted from *S. cerevisiae* SK-1 and pre-treated with Dotap (10 μl/μg of nucleic acids) for 30 min at 37°C without stimulation (Unst.) and with the laboratory strain of *S. cerevisiae* SK-1 at multiplicity of infection = 5 (SK-1/cDC). The levels of IL-6 were measured in the culture supernatants by ELISA. The graphs show the mean ± SEM of three independent experiments, each from different donors. One-way ANOVA was used to compare different experimental conditions (**p*-value ≤ 0.05).

Supplementary Figure 6 The quality and purity of DNA and RNA extracted from SK-1. The integrity of DNA and RNA samples isolated from SK-1 was analyzed by 1% agarose gel electrophoresis **(A)**. The measurements of absorbance 260/280 ratio of DNA and RNA samples were performed by Nanodrop spectrophotometer **(B)**.

Supplementary Figure 7 Gating strategy for the purification of human blood naïve CD4 T cells. Human peripheral blood mononuclear cells purified from the peripheral blood of healthy donors were labeled with specific antibodies conjugated with a fluorochrome and, by using a cell sorter, naïve CD4 T cells were isolated as CD4^high^, CD45RA^high^, CD45RO^-^, and CD27^+^ **(A)**. The purity of the isolated naïve CD4 T cells, evaluated by flow cytometry after purification, is more than 97% **(B)**. The plots show the data of a representative of all experiments performed with dendritic cells or naïve CD4 T cells.

Supplementary Figure 8 Expression of the yeast receptor in human plasmacytoid dendritic cells (pDCs). The expression values of yeast pattern recognition receptors, cell wall **(A)** and nucleic acid sensors **(B)**, on pDCs freshly isolated from the blood of healthy donors were extracted from Affymetrix data (Human Genome U133 Plus 2.0 arrays) (48). The dashed line is the threshold of signal detection. White bars represent the mRNA levels of the receptors expressed on pDCs; gray bars indicate that the receptors were not detectable. The data are the mean of seven independent experiments, each from different donors. The error bars represent standard deviation.
