# Supplementary figures and images for: Human Conventional and Plasmacytoid Dendritic Cells Differ in Their Ability to Respond to Saccharomyces cerevisiae
Source: Front Immunol. 2022 May 11;13:850404. doi: 10.3389/fimmu.2022.850404 (PMC9131191; doi:10.3389/fimmu.2022.850404)

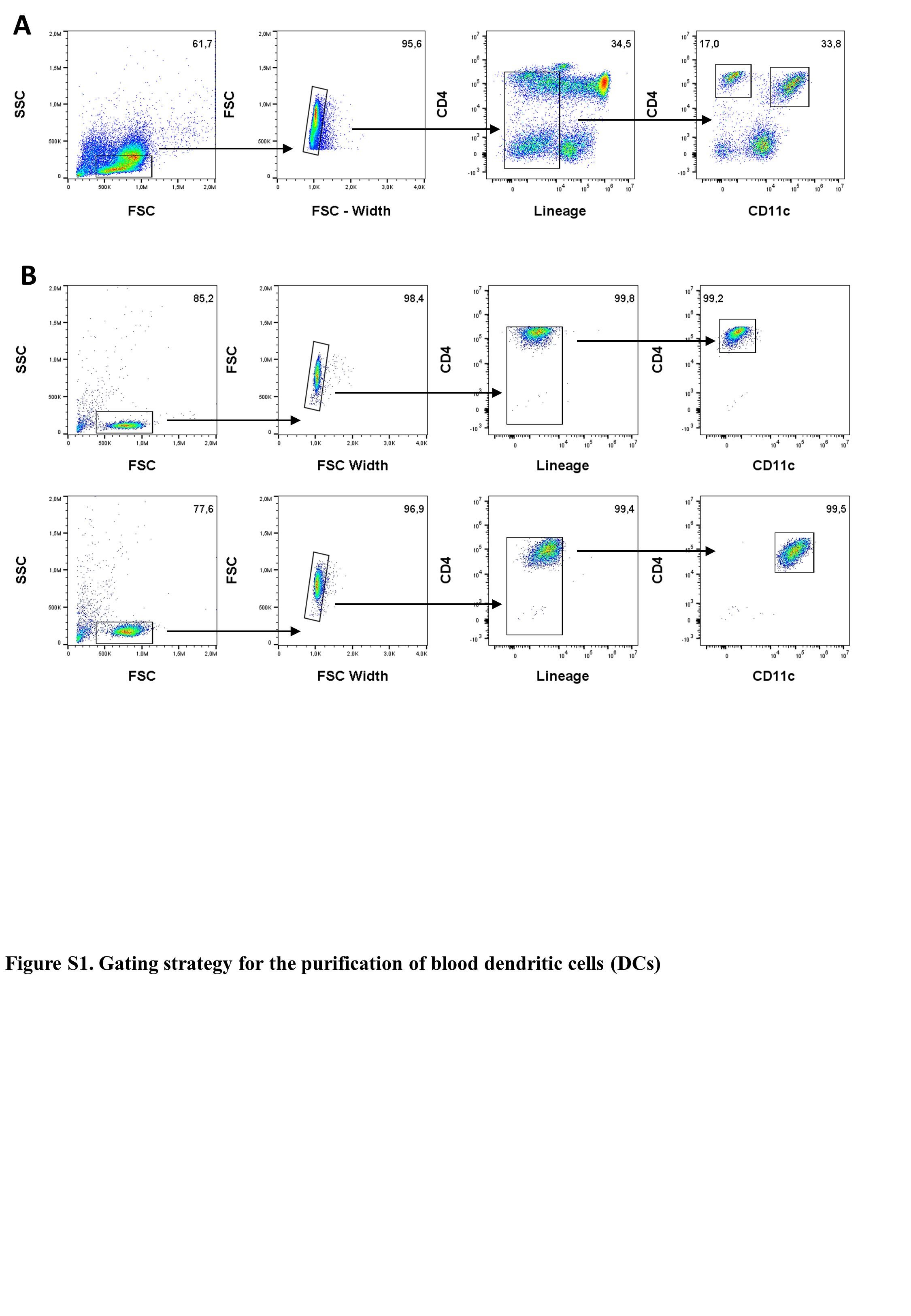

Supplement: Supplementary Figure 1 — Gating strategy for the purification of blood dendritic cells (DCs). Human peripheral blood mononuclear cells purified from the peripheral blood of healthy donors were enriched using the Human Pan-DC Pre-Enrichment Kit (EasySep) and then labeled with specific antibodies conjugated with a fluorochrome. Using a cell sorter, the plasmacytoid dendritic cells (pDCs) were isolated as lineage (CD3, CD14, CD16, CD19, CD56, CD235α)- CD4+, CD11c-, while conventional dendritic cells (cDCs) were isolated as lineage (CD3, CD14, CD16, CD19, CD56, CD235α)- CD4+, CD11c+ (A). The purity of the isolated pDCs and cDCs, evaluated by flow cytometry after purification, is more than 94% (B). [file Image_1.tif]

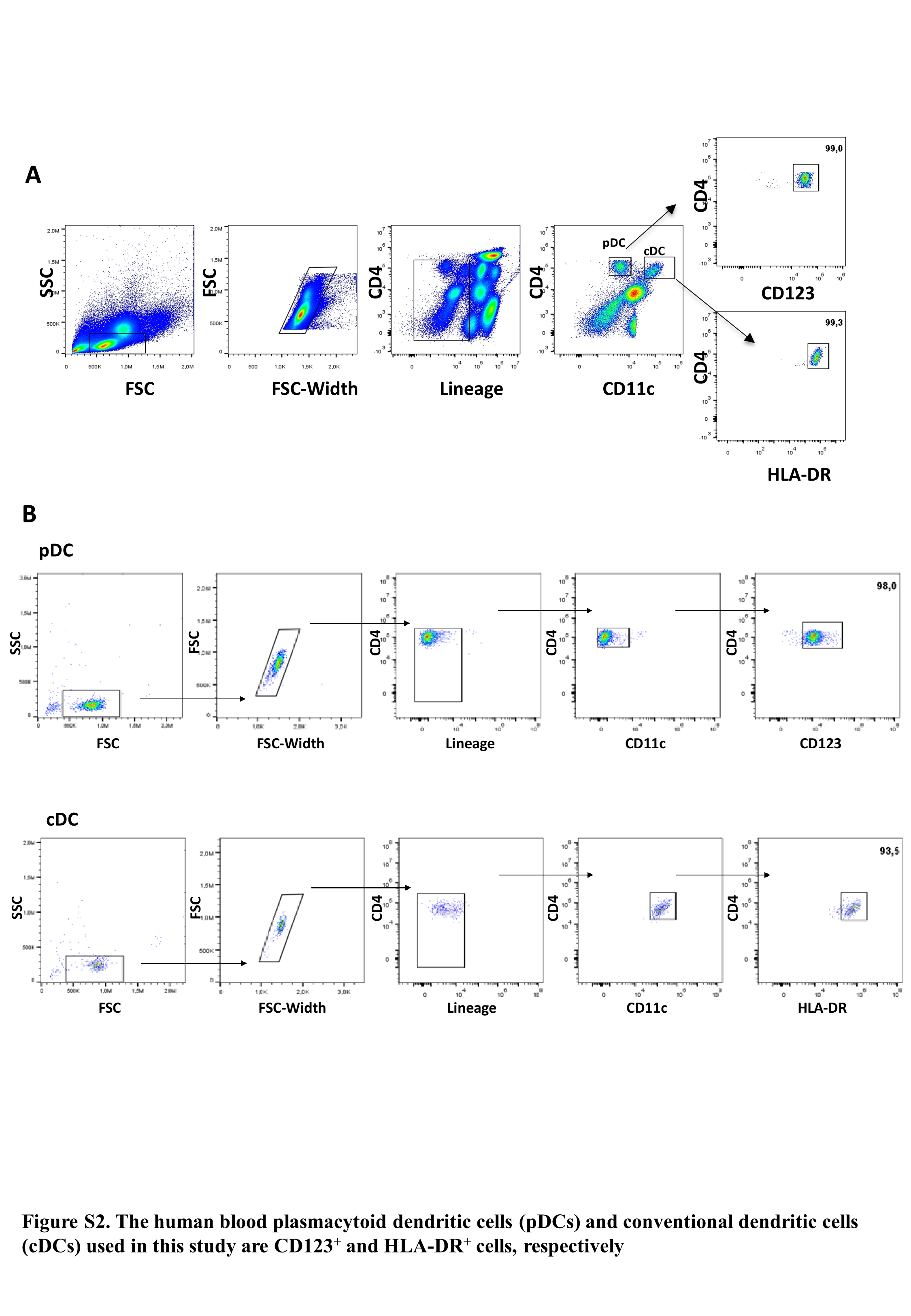

Supplement: Supplementary Figure 2 — The human blood plasmacytoid dendritic cells (pDCs) and conventional dendritic cells (cDCs) used in this study are CD123+ and HLA-DR+ cells, respectively. Human peripheral blood mononuclear cells purified from the peripheral blood of healthy donors were labeled with specific antibodies conjugated with a fluorochrome. pDCs were identified as lineage (CD3, CD14, CD16, CD19, CD56, CD235α)- CD4+, CD11c-, while cDCs were identified as lineage (CD3, CD14, CD16, CD19, CD56, CD235α)- CD4+, CD11c+. Staining of CD123 and HLA-DR before (A) and after sorting (B) reveals that pDCs are CD123+ cells, while cDCs are HLA-DR+ cells. The plots show the data of a representative of more experiments. [file Image_2.tif]

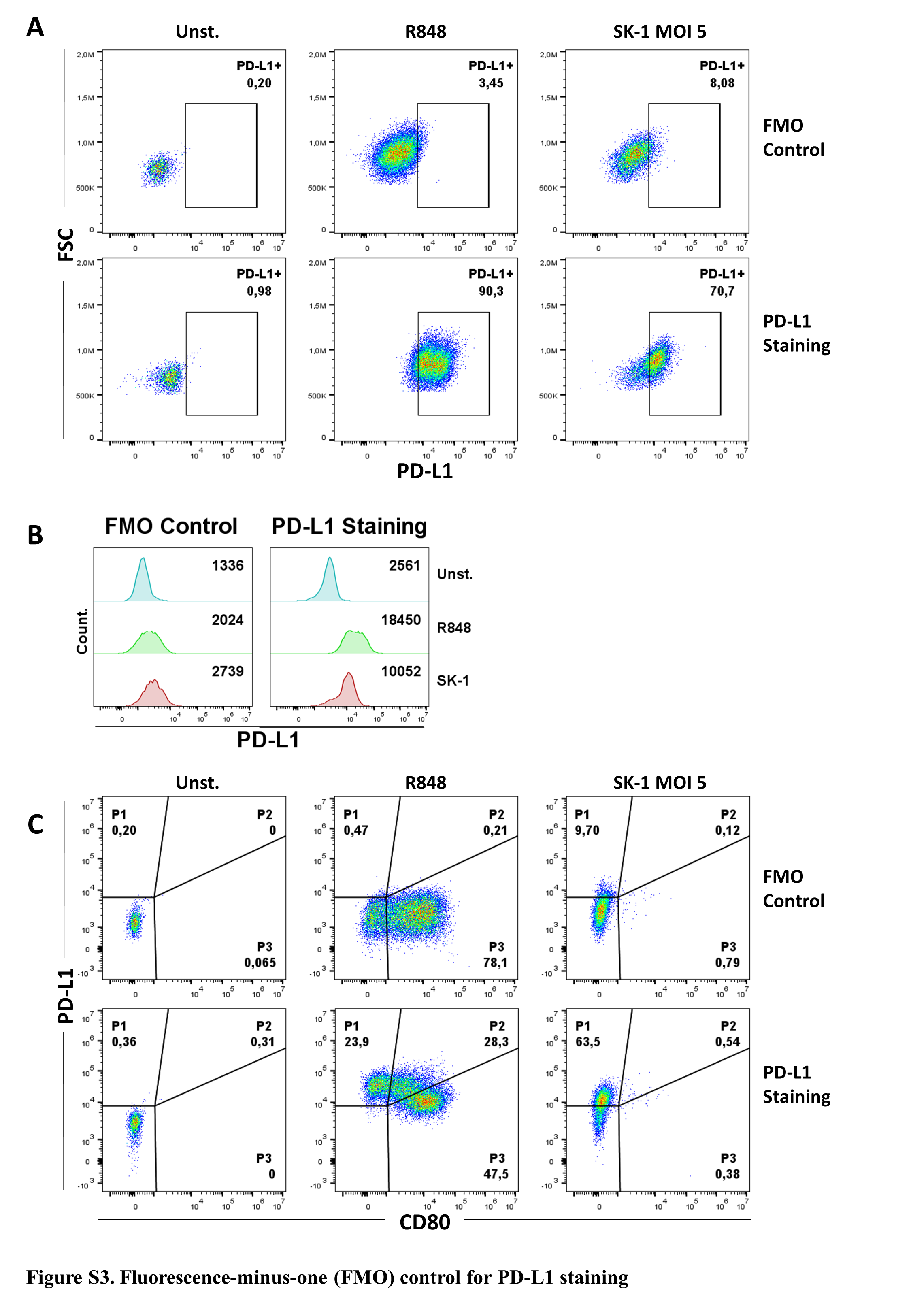

Supplement: Supplementary Figure 3 — Fluorescence-minus-one control (FMO) for PD-L1 staining. Human plasmacytoid dendritic cells (pDCs) purified from the peripheral blood of healthy donors were cultured for 48 h without stimulation (Unst.), with R848 (1 μg/ml), or with the laboratory strain of Saccharomyces cerevisiae SK-1 at multiplicity of infection = 5 (colony-forming unit SK-1/pDC). Cells were stained with anti-CD80 BV650 (FMO control) or anti-PD-L1 PE and anti-CD80 BV650 (PD-L1 staining). Percentage of PD-L1+ cells (A), MFI of PD-L1 (B), and percentage of P1, P2, P3 pDC subpopulations (C) were represented. Plots and histograms are from one representative of more experiments. [file Image_3.tif]

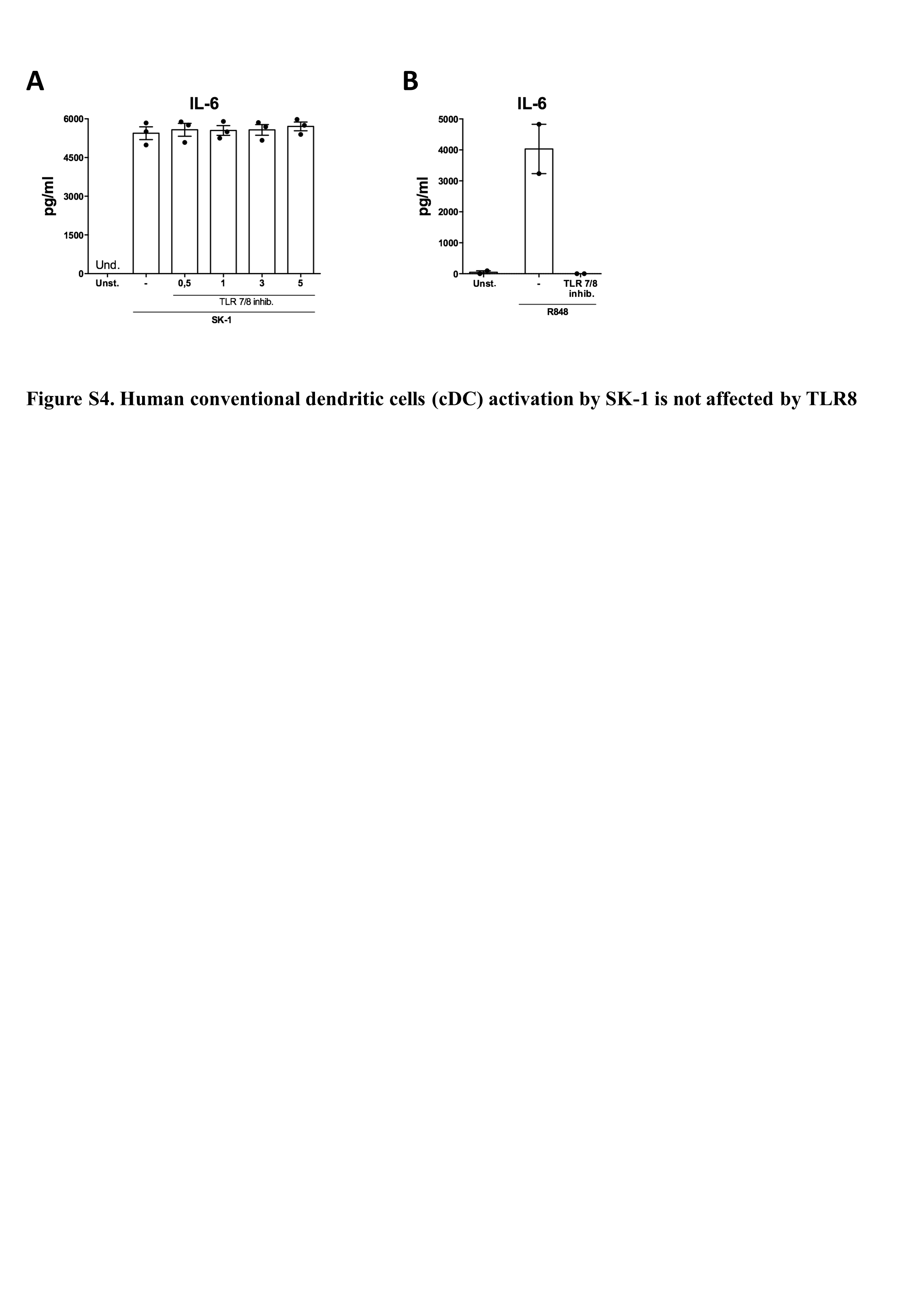

Supplement: Supplementary Figure 4 — Human conventional dendritic cell (cDC) activation by SK-1 is not affected by TLR8. Human cDCs purified from the peripheral blood of healthy donors were cultured for 48 h without stimulation (Unst.) and pre-treated for 30 min with different doses of TLR inhibitor, as indicated, with the laboratory strain of Saccharomyces cerevisiae SK-1 at multiplicity of infection = 5 (SK-1/conventional dendritic cells) (A), or with 5 μg/ml TLR8 inhibitor and R848 (0.1 μg/ml) (B). The levels of IL-6 were measured in the culture supernatants by ELISA. The graphs show the mean ± SEM of 3 independent experiments, each from different donors. Two-way ANOVA was used to compare different experimental conditions (*p-value ≤ 0.05). [file Image_4.tif]

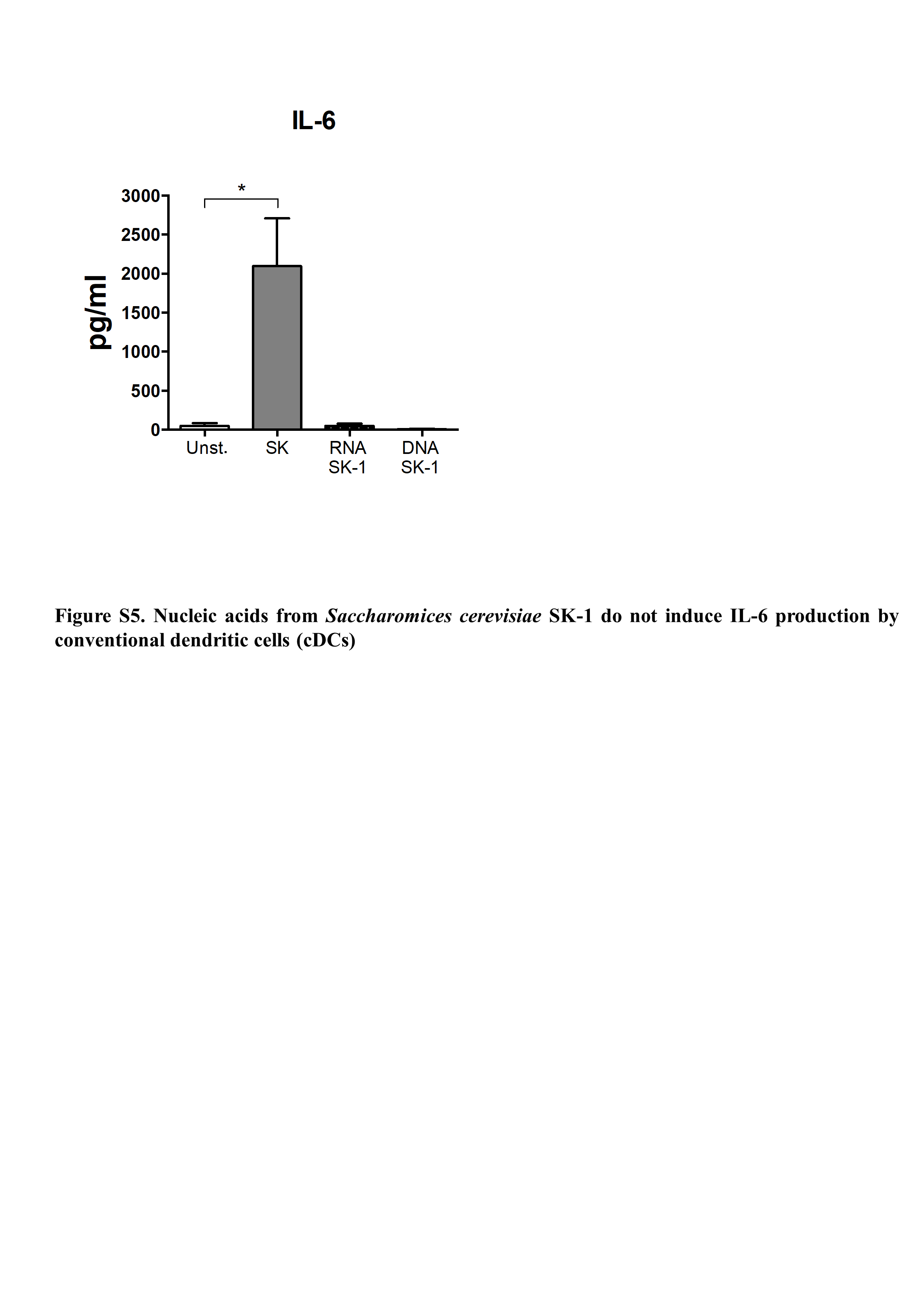

Supplement: Supplementary Figure 5 — Nucleic acids from Saccharomyces cerevisiae SK-1 do not induce the production of IL-6 by conventional dendritic cells (cDCs). Human cDCs purified from the peripheral blood of healthy donors were cultured for 48 h with RNA or DNA (0.2 μg) extracted from S. cerevisiae SK-1 and pre-treated with Dotap (10 μl/μg of nucleic acids) for 30 min at 37°C without stimulation (Unst.) and with the laboratory strain of S. cerevisiae SK-1 at multiplicity of infection = 5 (SK-1/cDC). The levels of IL-6 were measured in the culture supernatants by ELISA. The graphs show the mean ± SEM of three independent experiments, each from different donors. One-way ANOVA was used to compare different experimental conditions (*p-value ≤ 0.05). [file Image_5.tif]

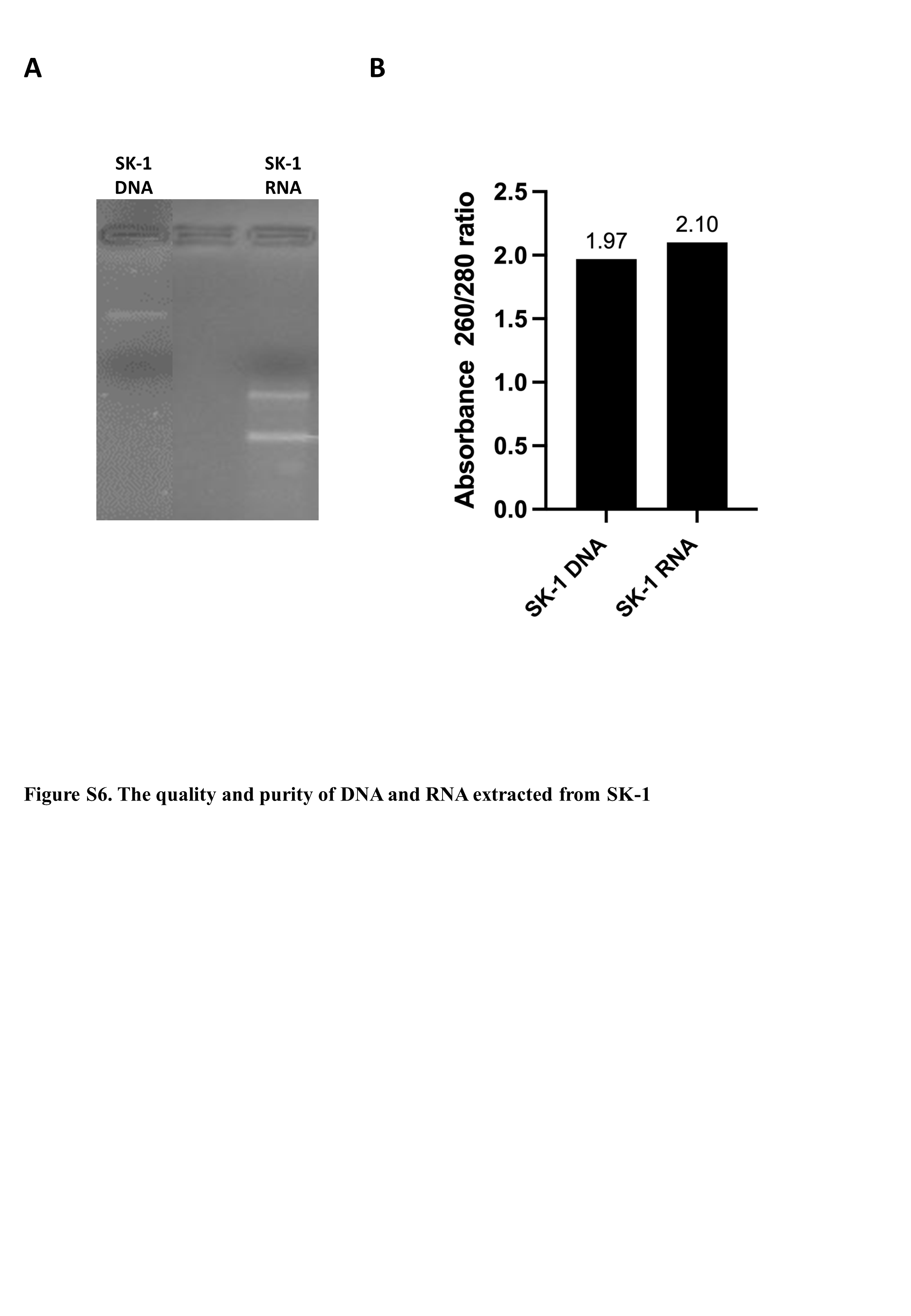

Supplement: Supplementary Figure 6 — The quality and purity of DNA and RNA extracted from SK-1. The integrity of DNA and RNA samples isolated from SK-1 was analyzed by 1% agarose gel electrophoresis (A). The measurements of absorbance 260/280 ratio of DNA and RNA samples were performed by Nanodrop spectrophotometer (B). [file Image_6.tif]

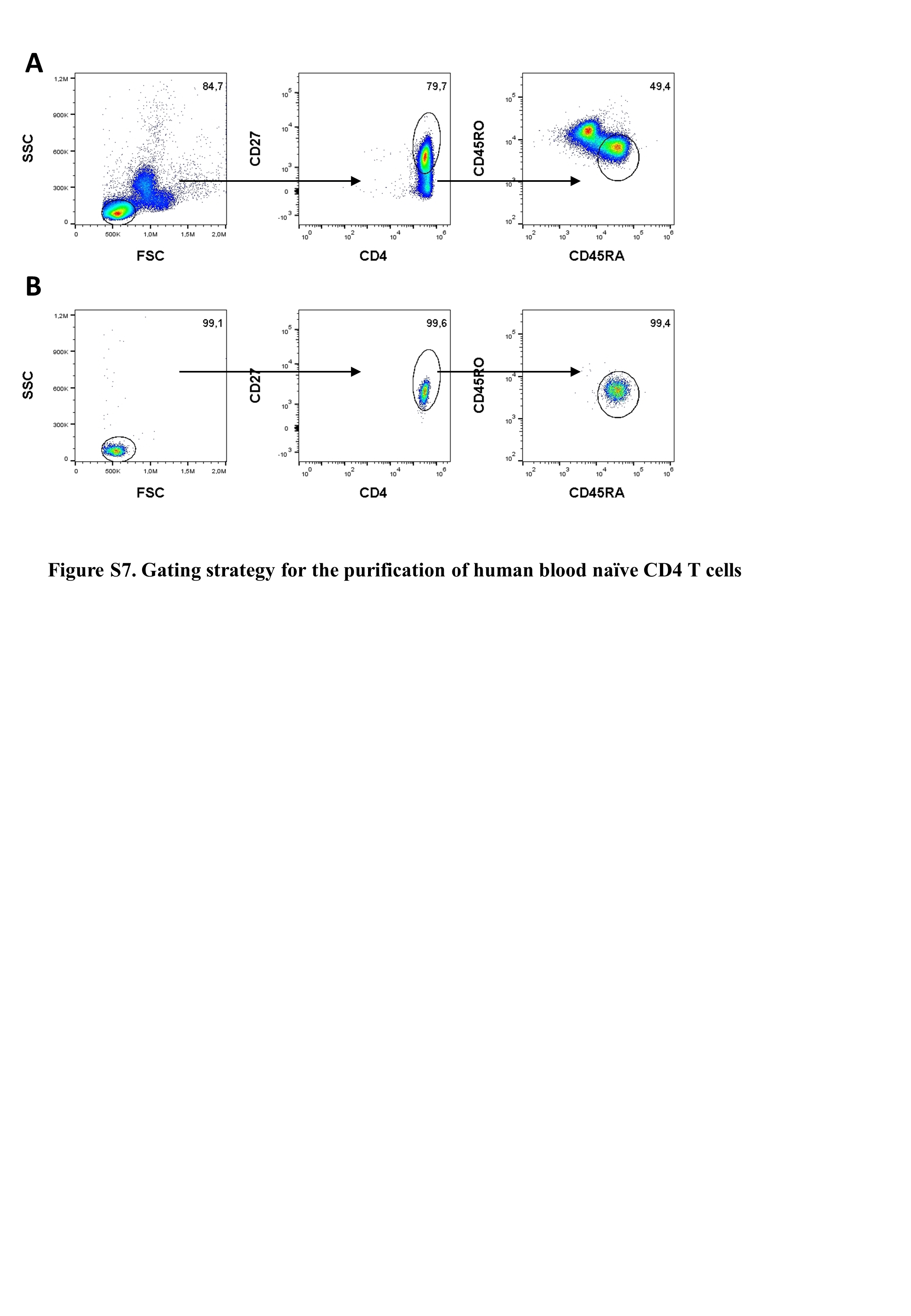

Supplement: Supplementary Figure 7 — Gating strategy for the purification of human blood naïve CD4 T cells. Human peripheral blood mononuclear cells purified from the peripheral blood of healthy donors were labeled with specific antibodies conjugated with a fluorochrome and, by using a cell sorter, naïve CD4 T cells were isolated as CD4high, CD45RAhigh, CD45RO-, and CD27+ (A). The purity of the isolated naïve CD4 T cells, evaluated by flow cytometry after purification, is more than 97% (B). The plots show the data of a representative of all experiments performed with dendritic cells or naïve CD4 T cells. [file Image_7.tif]

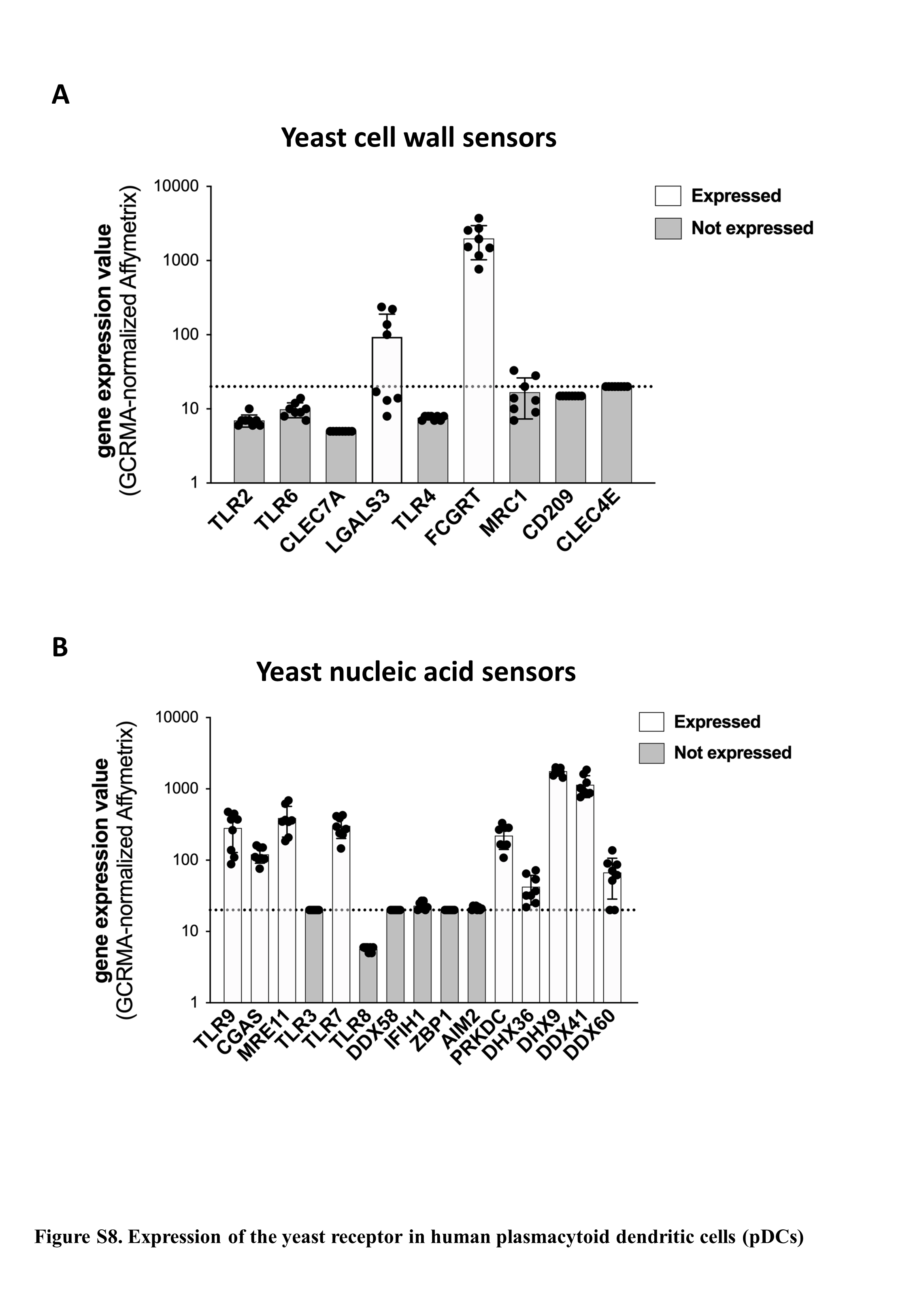

Supplement: Supplementary Figure 8 — Expression of the yeast receptor in human plasmacytoid dendritic cells (pDCs). The expression values of yeast pattern recognition receptors, cell wall (A) and nucleic acid sensors (B), on pDCs freshly isolated from the blood of healthy donors were extracted from Affymetrix data (Human Genome U133 Plus 2.0 arrays) (48). The dashed line is the threshold of signal detection. White bars represent the mRNA levels of the receptors expressed on pDCs; gray bars indicate that the receptors were not detectable. The data are the mean of seven independent experiments, each from different donors. The error bars represent standard deviation. [file Image_8.tif]
